# Diethyl 2-{[2-(trifluoro­meth­yl)anil­ino]methyl­idene}propane­dioate

**DOI:** 10.1107/S1600536812002590

**Published:** 2012-01-25

**Authors:** B. Garudachari, Arun M. Isloor, M. N. Satyanarayan, Thomas Gerber, Eric Hosten, Richard Betz

**Affiliations:** a National Institute of Technology-Karnataka, Department of Chemistry – Organic Electronics Division, Surathkal, Mangalore 575 025, India; b National Institute of Technology-Karnataka, Department of Physics, Surathkal, Mangalore 575 025, India; c Nelson Mandela Metropolitan University, Summerstrand Campus, Department of Chemistry, University Way, Summerstrand, PO Box 77000, Port Elizabeth 6031, South Africa

## Abstract

The title compound, C_15_H_16_F_3_NO_4_, is an *N*-substituted derivative of *ortho*-trifluoro­methyl­aniline featuring a twofold Michael system. The least-squares planes defined by the atoms of the phenyl ring and the atoms of the Michael system enclose an angle of 15.52 (5)°. Apart from classical intra­molecular N—H⋯O and N—H⋯F hydrogen bonds, inter­molecular C—H⋯O contacts are observed, the latter connecting the mol­ecules into chains along [110]. The shortest inter­centroid distance between two aromatic systems is 3.6875 (9) Å.

## Related literature

For the crystal structure of another *ortho*-trifluoro­methyl aniline derivative featuring a Michael system as substituent, see: Schweinfurth *et al.* (2011 ▶). For general information on Michael systems, see: McMurry (1992 ▶). For general pharmaceutical background to derivatives of the title compound, see: Kaur *et al.* (2010 ▶); Eswaran *et al.* (2010 ▶); Chou *et al.* (2010 ▶); Chen *et al.* (2004 ▶); Shingalapur *et al.* (2009 ▶). For the preparation of the title compound, see: Eswaran *et al.* (2009 ▶) For the graph-set analysis of hydrogen bonds, see: Etter *et al.* (1990 ▶); Bernstein *et al.* (1995 ▶).
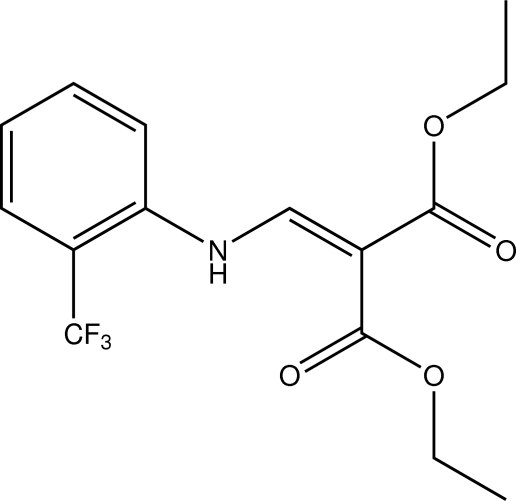


## Experimental

### 

#### Crystal data

C_15_H_16_F_3_NO_4_
*M* *_r_* = 331.29
Triclinic, 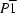

*a* = 7.8080 (2) Å
*b* = 10.1485 (3) Å
*c* = 10.5265 (3) Å
α = 95.193 (1)°
β = 109.183 (1)°
γ = 99.405 (1)°
*V* = 767.84 (4) Å^3^
*Z* = 2
Mo *K*α radiation
μ = 0.13 mm^−1^
*T* = 200 K
0.55 × 0.39 × 0.09 mm

#### Data collection

Bruker APEXII CCD diffractometer
Absorption correction: multi-scan (*SADABS*; Bruker, 2008 ▶) *T*_min_ = 0.946, *T*_max_ = 1.000
13616 measured reflections
3825 independent reflections
3240 reflections with *I* > 2σ(*I*)
*R*_int_ = 0.014

#### Refinement

*R*[*F* ^2^ > 2σ(*F* ^2^)] = 0.044
*wR*(*F* ^2^) = 0.128
*S* = 1.05
3825 reflections
210 parameters
H-atom parameters constrained
Δρ_max_ = 0.39 e Å^−3^
Δρ_min_ = −0.34 e Å^−3^

### 

Data collection: *APEX2* (Bruker, 2010 ▶); cell refinement: *SAINT* (Bruker, 2010 ▶); data reduction: *SAINT*; program(s) used to solve structure: *SIR97* (Altomare *et al.*, 1999 ▶); program(s) used to refine structure: *SHELXL97* (Sheldrick, 2008 ▶); molecular graphics: *ORTEP-3* (Farrugia, 1997 ▶) and *Mercury* (Macrae *et al.*, 2008 ▶); software used to prepare material for publication: *SHELXL97* and *PLATON* (Spek, 2009 ▶).

## Supplementary Material

Crystal structure: contains datablock(s) I, global. DOI: 10.1107/S1600536812002590/bg2441sup1.cif

Supplementary material file. DOI: 10.1107/S1600536812002590/bg2441Isup2.cdx

Structure factors: contains datablock(s) I. DOI: 10.1107/S1600536812002590/bg2441Isup3.hkl

Supplementary material file. DOI: 10.1107/S1600536812002590/bg2441Isup4.cml

Additional supplementary materials:  crystallographic information; 3D view; checkCIF report

## Figures and Tables

**Table 1:** Hydrogen-bond geometry (Å, °)

*D*—H⋯*A*	*D*—H	H⋯*A*	*D*⋯*A*	*D*—H⋯*A*
N1—H1⋯F2	0.88	2.35	2.9330 (15)	124
N1—H1⋯F3	0.88	2.45	2.9242 (15)	114
N1—H1⋯O3	0.88	1.99	2.6399 (14)	130
C4—H4⋯O1^i^	0.95	2.60	3.2766 (17)	129

Symmetry code: (i) 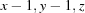.

**Fig. 1.**
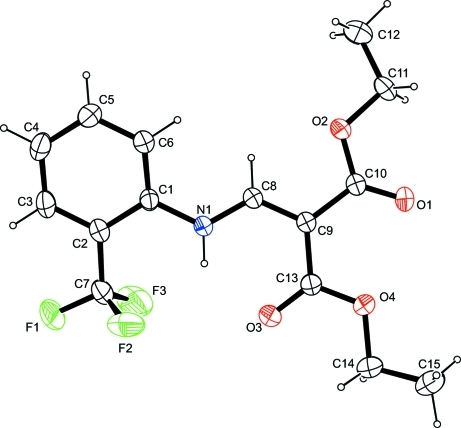
The molecular structure of the title compound, with atom labels and anisotropic displacement ellipsoids (drawn at 50% probability level).

**Fig. 2.**
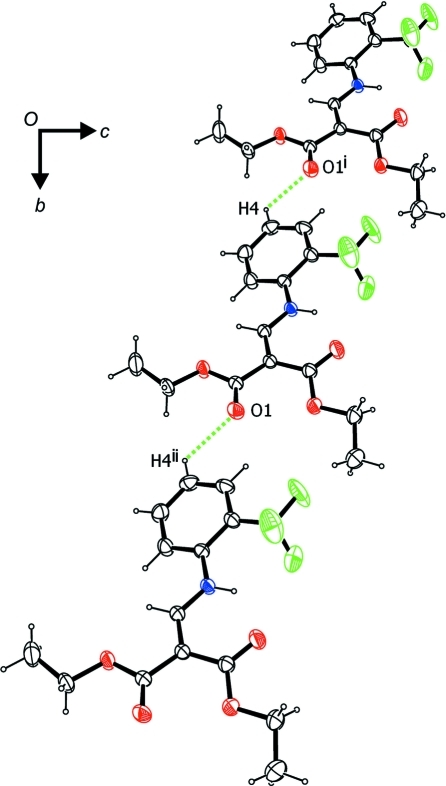
Intermolecular C–H···O contacts, viewed along [-1 0 0]. Symmetry operators: ix - 1, y - 1, z; iix + 1, y + 1, z.

## supplementary crystallographic information

### Comment

Esters of trifluoroaniline are very important intermediates to synthesize 8-fluoroquinoline derivatives, this moiety is of great importance to chemists as well as biologists as it is one of the key building elements for many naturally occurring compounds. Members of this family have a wide range of applications in pharmaceuticals as antimalarial (Kaur *et al.*, 2010), anti-tuberculosis (Eswaran *et al.*, 2010), antitumor (Chou *et al.*, 2010), anticancer (Chen *et al.*, 2004) and antiviral (Shingalapur *et al.*, 2009) agents. In view of the biological importance, we have synthesized the title compound to study its crystal structure.

Resonance between the aromatic system and the *N*-bonded twofold Michael system (an α,β-unsaturated carbonyl compound moiety, see for instance, McMurry, 1992) renders the carbon–nitrogen backbone of the molecule nearly planar, with the least-sqaures planes defined by the carbon atoms of the phenyl ring on the one hand and the non-hydrogen atoms of the twofold Michael system on the other hand enclosing an angle of only 15.52 (5) ° (Fig. 1). In the crystal, classical intramolecular hydrogen bridges of the N–H···O and N–H···F type can be observed between the secondary amine group and two of the fluorine atoms of the trifluoromethyl group and one of the double-bonded oxygen atoms. In addition, an intramolecular C–H···F contact (*d*_C···F_: 2.6805 (19) Å) involving one of the hydrogen atoms on the aromatic systems is obvious that explains the in-plane conformation of one of the trifluoromethyl group's fluorine atoms with the plane defined by the carbon atoms of the phenyl group. Intermolecular C–H···O contacts whose range falls by more than 0.1 Å below the sum of van-der-Waals radii of the atoms participating in them are present in the crystal structure. In terms of graph-set analysis (Etter *et al.*, 1990; Bernstein *et al.*, 1995), the descriptor for the latter type of contacts is *C*^1^_1_(10) on the unitary level. In total, the molecules are connected to infinite chains along [1 1 0]. Information about metrical parameters of these interactions can be found in Table 1. The shortest intercentroid distance between two aromatic systems was measured at 3.6875 (9) Å (Fig. 2).

### Experimental

A suspension of 2-(trifluoromethyl) aniline (1.0 g, 0.0062 mol) and diethyl(ethoxymethylene) malonate (4.02 g, 0.0186 mol) was heated to 110 °C for 4 h. The reaction mixture was cooled. The solid product obtained was filtered, washed with pet ether and recrystallized using ethanol. Yield: 1.81 g, 88.29%, m. p. 357–358 K, (Eswaran *et al.*, 2009).

### Refinement

Carbon-bound H atoms were placed in calculated positions (C—H 0.95 Å for aromatic and vinylic C atoms, C—H 0.99 Å for methylene groups) and were included in the refinement in the riding model approximation, with *U*(H) set to 1.2*U*_eq_(C). The H atoms of the methyl groups were allowed to rotate with a fixed angle around the C–C bond to best fit the experimental electron density (HFIX 137 in the *SHELX* program suite (Sheldrick, 2008)), with *U*(H) set to 1.5*U*_eq_(C). The nitrogen-bound H atom was placed in a calculated position (N—H 0.88 Å) and was included in the refinement in the riding model approximation, with *U*(H) set to 1.2*U*_eq_(N)

### Crystal data

**Table:**

C_15_H_16_F_3_NO_4_	*Z* = 2
*M**_r_* = 331.29	*F*(000) = 344
Triclinic, *P*1	*D*_x_ = 1.433 Mg m^−^^3^
Hall symbol: -P 1	Melting point: 357(1) K
*a* = 7.8080 (2) Å	Mo *K*α radiation, λ = 0.71073 Å
*b* = 10.1485 (3) Å	Cell parameters from 7916 reflections
*c* = 10.5265 (3) Å	θ = 2.8–28.3°
α = 95.193 (1)°	µ = 0.13 mm^−^^1^
β = 109.183 (1)°	*T* = 200 K
γ = 99.405 (1)°	Platelet, colourless
*V* = 767.84 (4) Å^3^	0.55 × 0.39 × 0.09 mm

### Data collection

**Table:**

Bruker APEXII CCD diffractometer	3825 independent reflections
Radiation source: fine-focus sealed tube	3240 reflections with *I* > 2σ(*I*)
graphite	*R*_int_ = 0.014
φ and ω scans	θ_max_ = 28.4°, θ_min_ = 2.1°
Absorption correction: multi-scan (*SADABS*; Bruker, 2008)	*h* = −10→10
*T*_min_ = 0.946, *T*_max_ = 1.000	*k* = −13→13
13616 measured reflections	*l* = −14→14

### Refinement

**Table:**

Refinement on *F*^2^	Primary atom site location: structure-invariant direct methods
Least-squares matrix: full	Secondary atom site location: difference Fourier map
*R*[*F*^2^ > 2σ(*F*^2^)] = 0.044	Hydrogen site location: inferred from neighbouring sites
*wR*(*F*^2^) = 0.128	H-atom parameters constrained
*S* = 1.05	*w* = 1/[σ^2^(*F*_o_^2^) + (0.0646*P*)^2^ + 0.2686*P*] where *P* = (*F*_o_^2^ + 2*F*_c_^2^)/3
3825 reflections	(Δ/σ)_max_ < 0.001
210 parameters	Δρ_max_ = 0.39 e Å^−^^3^
0 restraints	Δρ_min_ = −0.33 e Å^−^^3^

### Fractional atomic coordinates and isotropic or equivalent isotropic displacement parameters (Å^2^)

**Table:**

	*x*	*y*	*z*	*U*_iso_*/*U*_eq_	
F1	0.3636 (2)	−0.04352 (13)	0.35986 (11)	0.0857 (5)	
F2	0.3880 (2)	0.16548 (13)	0.34858 (11)	0.0744 (4)	
F3	0.60489 (16)	0.07240 (15)	0.33735 (11)	0.0755 (4)	
O1	0.86819 (16)	0.61635 (10)	0.03165 (10)	0.0418 (3)	
O2	0.68442 (13)	0.44254 (9)	−0.12584 (9)	0.0335 (2)	
O3	0.71583 (16)	0.42107 (11)	0.32438 (10)	0.0431 (3)	
O4	0.82996 (15)	0.61975 (10)	0.27787 (9)	0.0374 (2)	
N1	0.49938 (14)	0.23376 (10)	0.11834 (10)	0.0264 (2)	
H1	0.5378	0.2582	0.2071	0.032*	
C1	0.36699 (16)	0.11270 (12)	0.06191 (12)	0.0251 (2)	
C2	0.32968 (18)	0.02013 (13)	0.14539 (13)	0.0295 (3)	
C3	0.20078 (19)	−0.10035 (13)	0.08848 (15)	0.0345 (3)	
H3	0.1757	−0.1621	0.1459	0.041*	
C4	0.10908 (19)	−0.13115 (13)	−0.05021 (15)	0.0360 (3)	
H4	0.0239	−0.2147	−0.0890	0.043*	
C5	0.1430 (2)	−0.03872 (15)	−0.13194 (15)	0.0376 (3)	
H5	0.0788	−0.0587	−0.2275	0.045*	
C6	0.26878 (19)	0.08245 (14)	−0.07735 (13)	0.0335 (3)	
H6	0.2882	0.1454	−0.1353	0.040*	
C7	0.4211 (2)	0.05075 (16)	0.29631 (15)	0.0439 (4)	
C8	0.57087 (16)	0.31427 (12)	0.04683 (12)	0.0247 (2)	
H8	0.5348	0.2837	−0.0480	0.030*	
C9	0.69146 (16)	0.43685 (12)	0.09707 (12)	0.0246 (2)	
C10	0.75905 (16)	0.51016 (12)	0.00254 (12)	0.0263 (2)	
C11	0.7483 (2)	0.50110 (15)	−0.22660 (14)	0.0353 (3)	
H11A	0.8853	0.5197	−0.1952	0.042*	
H11B	0.7065	0.5872	−0.2421	0.042*	
C12	0.6691 (3)	0.4020 (2)	−0.35413 (17)	0.0588 (5)	
H12A	0.5338	0.3800	−0.3810	0.088*	
H12B	0.7180	0.3196	−0.3391	0.088*	
H12C	0.7030	0.4411	−0.4265	0.088*	
C13	0.74669 (17)	0.48967 (13)	0.24184 (13)	0.0287 (3)	
C14	0.8920 (2)	0.67197 (15)	0.42245 (14)	0.0432 (4)	
H14A	1.0000	0.6351	0.4733	0.052*	
H14B	0.7915	0.6459	0.4588	0.052*	
C15	0.9441 (4)	0.82171 (18)	0.43692 (19)	0.0652 (6)	
H15A	1.0332	0.8459	0.3913	0.098*	
H15B	1.0003	0.8600	0.5337	0.098*	
H15C	0.8332	0.8578	0.3953	0.098*	

### Atomic displacement parameters (Å^2^)

**Table:**

	*U*^11^	*U*^22^	*U*^33^	*U*^12^	*U*^13^	*U*^23^
F1	0.1160 (11)	0.0754 (8)	0.0410 (6)	−0.0340 (7)	0.0158 (6)	0.0275 (6)
F2	0.1112 (10)	0.0703 (8)	0.0401 (6)	0.0085 (7)	0.0324 (6)	−0.0040 (5)
F3	0.0501 (6)	0.1190 (11)	0.0426 (6)	0.0030 (6)	−0.0001 (5)	0.0263 (6)
O1	0.0542 (6)	0.0302 (5)	0.0357 (5)	−0.0117 (4)	0.0186 (5)	0.0039 (4)
O2	0.0392 (5)	0.0344 (5)	0.0256 (4)	−0.0053 (4)	0.0160 (4)	0.0038 (4)
O3	0.0599 (7)	0.0368 (5)	0.0243 (5)	−0.0072 (5)	0.0115 (4)	0.0062 (4)
O4	0.0508 (6)	0.0286 (5)	0.0254 (5)	−0.0056 (4)	0.0111 (4)	0.0000 (4)
N1	0.0288 (5)	0.0256 (5)	0.0226 (5)	−0.0010 (4)	0.0092 (4)	0.0048 (4)
C1	0.0255 (5)	0.0230 (5)	0.0274 (6)	0.0017 (4)	0.0115 (4)	0.0046 (4)
C2	0.0320 (6)	0.0278 (6)	0.0307 (6)	0.0035 (5)	0.0137 (5)	0.0088 (5)
C3	0.0378 (7)	0.0252 (6)	0.0434 (7)	0.0022 (5)	0.0186 (6)	0.0106 (5)
C4	0.0340 (6)	0.0251 (6)	0.0459 (8)	−0.0017 (5)	0.0156 (6)	−0.0011 (5)
C5	0.0372 (7)	0.0377 (7)	0.0313 (7)	−0.0039 (6)	0.0108 (5)	−0.0015 (5)
C6	0.0350 (6)	0.0341 (7)	0.0278 (6)	−0.0034 (5)	0.0112 (5)	0.0058 (5)
C7	0.0511 (8)	0.0435 (8)	0.0324 (7)	−0.0059 (6)	0.0140 (6)	0.0135 (6)
C8	0.0260 (5)	0.0246 (5)	0.0245 (5)	0.0034 (4)	0.0108 (4)	0.0051 (4)
C9	0.0265 (5)	0.0236 (5)	0.0239 (6)	0.0030 (4)	0.0098 (4)	0.0050 (4)
C10	0.0282 (5)	0.0247 (5)	0.0270 (6)	0.0036 (4)	0.0111 (5)	0.0061 (4)
C11	0.0411 (7)	0.0387 (7)	0.0315 (7)	0.0042 (6)	0.0197 (6)	0.0133 (5)
C12	0.0817 (13)	0.0610 (11)	0.0340 (8)	−0.0048 (9)	0.0308 (9)	0.0038 (7)
C13	0.0301 (6)	0.0277 (6)	0.0254 (6)	0.0014 (5)	0.0080 (5)	0.0046 (5)
C14	0.0556 (9)	0.0387 (8)	0.0251 (6)	−0.0028 (6)	0.0083 (6)	−0.0011 (5)
C15	0.1017 (16)	0.0404 (9)	0.0358 (8)	−0.0061 (9)	0.0137 (9)	−0.0067 (7)

### Geometric parameters (Å, °)

**Table:**

F1—C7	1.3179 (16)	C5—C6	1.3832 (18)
F2—C7	1.342 (2)	C5—H5	0.9500
F3—C7	1.329 (2)	C6—H6	0.9500
O1—C10	1.2042 (15)	C8—C9	1.3728 (16)
O2—C10	1.3492 (15)	C8—H8	0.9500
O2—C11	1.4464 (14)	C9—C13	1.4630 (17)
O3—C13	1.2207 (16)	C9—C10	1.4741 (16)
O4—C13	1.3327 (15)	C11—C12	1.484 (2)
O4—C14	1.4551 (16)	C11—H11A	0.9900
N1—C8	1.3357 (14)	C11—H11B	0.9900
N1—C1	1.4071 (15)	C12—H12A	0.9800
N1—H1	0.8800	C12—H12B	0.9800
C1—C6	1.3908 (17)	C12—H12C	0.9800
C1—C2	1.4029 (16)	C14—C15	1.488 (2)
C2—C3	1.3913 (18)	C14—H14A	0.9900
C2—C7	1.4909 (19)	C14—H14B	0.9900
C3—C4	1.378 (2)	C15—H15A	0.9800
C3—H3	0.9500	C15—H15B	0.9800
C4—C5	1.380 (2)	C15—H15C	0.9800
C4—H4	0.9500		
			
C10—O2—C11	116.56 (10)	C8—C9—C10	118.54 (11)
C13—O4—C14	115.84 (10)	C13—C9—C10	122.74 (10)
C8—N1—C1	124.71 (10)	O1—C10—O2	122.13 (11)
C8—N1—H1	117.6	O1—C10—C9	126.34 (12)
C1—N1—H1	117.6	O2—C10—C9	111.52 (10)
C6—C1—C2	118.40 (11)	O2—C11—C12	107.34 (12)
C6—C1—N1	120.99 (11)	O2—C11—H11A	110.2
C2—C1—N1	120.61 (11)	C12—C11—H11A	110.2
C3—C2—C1	120.22 (12)	O2—C11—H11B	110.2
C3—C2—C7	118.76 (12)	C12—C11—H11B	110.2
C1—C2—C7	120.98 (11)	H11A—C11—H11B	108.5
C4—C3—C2	120.77 (12)	C11—C12—H12A	109.5
C4—C3—H3	119.6	C11—C12—H12B	109.5
C2—C3—H3	119.6	H12A—C12—H12B	109.5
C3—C4—C5	118.95 (12)	C11—C12—H12C	109.5
C3—C4—H4	120.5	H12A—C12—H12C	109.5
C5—C4—H4	120.5	H12B—C12—H12C	109.5
C4—C5—C6	121.24 (13)	O3—C13—O4	121.74 (12)
C4—C5—H5	119.4	O3—C13—C9	122.85 (11)
C6—C5—H5	119.4	O4—C13—C9	115.40 (10)
C5—C6—C1	120.37 (12)	O4—C14—C15	107.04 (12)
C5—C6—H6	119.8	O4—C14—H14A	110.3
C1—C6—H6	119.8	C15—C14—H14A	110.3
F1—C7—F3	108.38 (14)	O4—C14—H14B	110.3
F1—C7—F2	105.40 (15)	C15—C14—H14B	110.3
F3—C7—F2	103.63 (14)	H14A—C14—H14B	108.6
F1—C7—C2	113.24 (13)	C14—C15—H15A	109.5
F3—C7—C2	113.39 (13)	C14—C15—H15B	109.5
F2—C7—C2	112.06 (13)	H15A—C15—H15B	109.5
N1—C8—C9	126.33 (11)	C14—C15—H15C	109.5
N1—C8—H8	116.8	H15A—C15—H15C	109.5
C9—C8—H8	116.8	H15B—C15—H15C	109.5
C8—C9—C13	118.72 (10)		
			
C8—N1—C1—C6	−14.70 (19)	C1—C2—C7—F2	57.59 (18)
C8—N1—C1—C2	165.95 (11)	C1—N1—C8—C9	175.78 (12)
C6—C1—C2—C3	1.70 (19)	N1—C8—C9—C13	−1.73 (19)
N1—C1—C2—C3	−178.93 (11)	N1—C8—C9—C10	178.62 (11)
C6—C1—C2—C7	−175.94 (13)	C11—O2—C10—O1	2.65 (19)
N1—C1—C2—C7	3.43 (19)	C11—O2—C10—C9	−176.71 (10)
C1—C2—C3—C4	0.5 (2)	C8—C9—C10—O1	−177.98 (13)
C7—C2—C3—C4	178.20 (13)	C13—C9—C10—O1	2.4 (2)
C2—C3—C4—C5	−1.9 (2)	C8—C9—C10—O2	1.34 (16)
C3—C4—C5—C6	1.0 (2)	C13—C9—C10—O2	−178.29 (11)
C4—C5—C6—C1	1.2 (2)	C10—O2—C11—C12	172.35 (13)
C2—C1—C6—C5	−2.6 (2)	C14—O4—C13—O3	3.8 (2)
N1—C1—C6—C5	178.08 (12)	C14—O4—C13—C9	−177.67 (12)
C3—C2—C7—F1	−1.0 (2)	C8—C9—C13—O3	12.48 (19)
C1—C2—C7—F1	176.66 (14)	C10—C9—C13—O3	−167.89 (13)
C3—C2—C7—F3	123.02 (15)	C8—C9—C13—O4	−166.01 (11)
C1—C2—C7—F3	−59.31 (19)	C10—C9—C13—O4	13.62 (18)
C3—C2—C7—F2	−120.09 (15)	C13—O4—C14—C15	−168.58 (15)

### Hydrogen-bond geometry (Å, °)

**Table:**

*D*—H···*A*	*D*—H	H···*A*	*D*···*A*	*D*—H···*A*
N1—H1···F2	0.88	2.35	2.9330 (15)	124
N1—H1···F3	0.88	2.45	2.9242 (15)	114
N1—H1···O3	0.88	1.99	2.6399 (14)	130
C3—H3···F1	0.95	2.33	2.6805 (19)	101
C4—H4···O1^i^	0.95	2.60	3.2766 (17)	129

Symmetry codes: (i) *x*−1, *y*−1, *z*.
